# Persistence barcodes: A novel approach to reducing bias in radiological analysis

**DOI:** 10.18632/oncotarget.28667

**Published:** 2024-11-12

**Authors:** Yashbir Singh, Colleen Farrelly, Quincy A. Hathaway, Gunnar Carlsson

**Keywords:** persistence barcodes, radiology, image features

## Abstract

Persistence barcodes emerge as a promising tool in radiological analysis, offering a novel approach to reduce bias and uncover hidden patterns in medical imaging. By leveraging topological data analysis, this technique provides a robust, multi-scale perspective on image features, potentially overcoming limitations in traditional methods and Graph Neural Networks. While challenges in interpretation and implementation remain, persistence barcodes show significant potential for improving diagnostic accuracy, standardization, and ultimately, patient outcomes in the evolving field of radiology.

## INTRODUCTION

In the ever-evolving landscape of medical imaging and radiological analysis, the quest for more accurate and unbiased diagnostic tools continues. As we push the boundaries of artificial intelligence (AI) and machine learning in healthcare, a promising technique from the field of topological data analysis (TDA) has emerged: persistence barcodes. This approach offers a fresh perspective on reducing bias in radiological analysis and uncovering patterns that traditional methods might overlook.

### Understanding persistence barcodes

Persistence barcodes are a visual representation of topological features in data across different scales [1]. They capture the birth, persistence, and death of features such as connected components, loops, and voids as the scale of observation changes. In the context of radiological images, these features could represent structures like tumors, blood vessels, or tissue densities [2].

The power of persistence barcodes lies in their ability to distill complex, high-dimensional data into a simple, interpretable format. This makes them particularly valuable in medical imaging, where the subtleties of image features can have significant diagnostic implications [3].

### Applications in radiological image analysis

When applied to radiological images, persistence barcodes can reveal patterns and structures that might be missed by traditional analysis methods. For instance, they can capture the intricate branching patterns of blood vessels or the subtle changes in tissue density that might indicate the early stages of a disease [4]. One of the key advantages of using persistence barcodes in radiological analysis is their potential to uncover hidden biases. By providing a topology-based view of the image data, they offer a perspective that is less influenced by the limitations of conventional image processing techniques or human visual perception [5]. As an example, by leveraging TDA’s ability to identify subtle changes in morphology, changing characteristics of adenocarcinoma spectrum lesions in the lung (i.e., ground glass and solid components) could be more acutely captured [6].

### Addressing GNN oversmoothing issues

Graph Neural Networks (GNNs) have shown promise in medical image analysis, but they often suffer from the problem of oversmoothing, where node features become indistinguishable after multiple layers [7]. While some recent advances in GNNs, such as sheaf neural networks, offer potential solutions to oversmoothing, they currently do not have as extensive prior study as persistence barcodes, which offer a well-tested solution to this issue [7].

By capturing topological features at various scales, persistence barcodes can preserve important structural information that might be lost during the smoothing process of GNNs. This multi-scale approach allows for a more nuanced analysis of the image data, potentially leading to more accurate and less biased results [8].

### Filtration advantage: Mitigating noise and instrument error

One of the standout features of persistence barcodes is their robustness to noise and small perturbations in the data [9]. This is particularly valuable in medical imaging, where instrument error or image artifacts can significantly impact analysis results.

The filtration process used to generate persistence barcodes allows for a systematic way to distinguish between persistent features (likely to be genuine structures) and transient ones (likely to be noise or artifacts). This built-in noise reduction mechanism can help mitigate biases that might arise from equipment-specific errors or variations in image quality [10].

### Comparing barcodes: A tool for standardization

Persistence barcodes offer a unique opportunity for standardization in radiological analysis. By comparing barcodes generated from images taken with different equipment or analyzed by different radiologists, we can identify and quantify variations that might indicate biases [11].

This comparative approach can be particularly powerful in:

- Calibrating equipment: By analyzing the persistence barcodes of standard test images across different machines, we can identify and correct for equipment-specific biases [12].
- Standardizing human interpretation: Comparing the barcodes of images analyzed by different radiologists can help identify individual biases and guide efforts towards more consistent interpretation standards [13].
- Longitudinal studies: In long-term studies, persistence barcodes can help account for changes in imaging technology or analysis techniques over time, ensuring consistency in data interpretation [14].

### Challenges and future directions

While persistence barcodes show great promise in reducing bias in radiological analysis, there are challenges to overcome. These include:

- Interpretation complexity: While barcodes are more interpretable than raw image data, they still require expertise to analyze effectively. Developing intuitive visualization tools and training programs will be crucial for widespread adoption [15].
- Computational demands: Generating persistence barcodes for high-resolution medical images can be computationally intensive. Advances in algorithms and hardware will be necessary to make this approach feasible for real-time clinical use [16].
- Integration with existing workflows: Incorporating persistence barcode analysis into established radiological workflows will require careful planning and validation to ensure it complements rather than disrupts current practices [17].

## CONCLUSIONS

Persistence barcodes represent a promising new frontier in the quest to reduce bias in radiological analysis. By offering a topology-based perspective on medical images, they have the potential to uncover patterns and structures that traditional methods might miss. Their robustness to noise, ability to capture multi-scale features, and potential for standardization make them a powerful tool in the radiologist’s arsenal.

As we continue to refine and validate this approach, persistence barcodes could play a crucial role in developing more accurate, consistent, and unbiased diagnostic tools. This, in turn, has the potential to improve patient outcomes and advance the field of radiology as a whole.
